# Transcriptome dynamics during cholesterol-induced transdifferentiation of human coronary artery smooth muscle cells: A Gene Ontology-centric clustering approach

**DOI:** 10.1016/j.bbrep.2021.101061

**Published:** 2021-06-27

**Authors:** Kentaro Inoue, Hiromitsu Araki, Fumihito Miura, Takashi Ito

**Affiliations:** a Department of Biochemistry, Kyushu University Graduate School of Medical Sciences, Fukuoka, Japan; b Department of Surgery and Science, Kyushu University Graduate School of Medical Sciences, Fukuoka, Japan

**Keywords:** Smooth muscle cell, Cholesterol, Epigenetics, Inflammation, Dedifferentiation

## Abstract

Macrophage-like cells derived from vascular smooth muscle cells (SMCs) play critical roles in atherogenesis, and DNA hydroxymethylation was implicated in transdifferentiation. We examined transcriptomes and (hydroxy)methylomes of human coronary artery SMCs during cholesterol-induced transdifferentiation. A unique approach of exhaustive identification of differentially expressed genes followed by Gene Ontology-centric clustering facilitated deeper understanding of multifaceted modulations of genes involved in extracellular matrix organization, angiogenesis, cell migration, hypoxia response, and cholesterol biosynthesis. Intriguingly, type I interferon response was transiently activated, presumably forming an immuno-metabolic circuit with cholesterol metabolism. Neither global nor DEG-proximal changes were evident in (hydroxy)methylation. These results would not only provide a unique data resource for atherosclerosis research but present a potentially useful approach in transcriptome data interpretation.

## Highlights

• Cholesterol-induced transcriptome dynamics revealed in human vascular SMCs.
• Type I interferon response transiently activated in cholesterol-loaded SMCs.
• Interpretation of transcriptome dynamics facilitated by GO-centric clustering.

## 1 Introduction

Atherosclerosis following hypercholesterolemia has remained as an important clinical issue even today [[1], [2], [3]], and explications of its mechanism are still essential considerations. For many years, pathogenesis of atherosclerosis has been characterized by chronic inflammation and lipid accumulation in the vasculature with a focus on macrophages [[4], [5], [6], [7], [8]]. However, a role for vascular smooth muscle cells (SMCs) in the development of atherosclerosis has been attracting more intense attention than ever before. This is primarily because it becomes increasingly evident that the contribution of SMCs to atherosclerotic plaque pathogenesis has been underestimated due to the SMC's phenotypic plasticity, including its ability to mimic macrophages. The results of coimmunostaining with the macrophage marker CD68 and the SMC marker α-smooth muscle actin (ASMA) suggested that 40% of CD68-positive cells in advanced human coronary atherosclerotic lesions could be originated from SMCs [9]. More importantly, Feil et al. described that SMCs can convert to macrophage-like cells during advancement of atherosclerotic lesions, losing the expression of classical SMC marker such as ASMA while turning positive for macrophage markers such as MAC-2 and CD68 [10]. Similarly, Shankman et al. not only found that more than 80% of SMCs within atherosclerotic lesions were ASMA negative but demonstrated the expression of markers of macrophages (e.g., LGALS3) in phenotypically modulated SMCs within lesions using SMC-traceable model mice [11]. These SMC-derived cells occupied up to one-third of total LGALS3-positive cells within advanced atherosclerotic lesions. These findings reinforce the importance of revealing the SMC-specific changes in atherosclerotic lesions independently of other cells such as macrophage and endothelial cells.

In experimental procedures to address atherosclerotic changes in SMCs in vitro, PDGF and cholesterol loading have been widely used as effective inducers. Cultured mouse aortic SMCs are known to conduct transdifferentiation to a macrophage-like state, or so-called “phenotype switching”, after cholesterol loading [[12], [13], [14], [15], [16]]. On the other hand, dynamics of transcriptome alterations during the phenotype switching of human SMCs has remained elusive. This study thus examined the transcriptome dynamics during cholesterol-induced transdifferentiation of human coronary artery SMCs (hcSMCs). We then analyzed the data using a unique Gene Ontology (GO)-centric clustering, which may complement the pitfalls of conventional approaches to maximize biological implications obtained from time course transcriptome data.

## 2 Material and methods

### 2.1 Cell culture and cholesterol loading

This study used hcSMCs that were obtained from Gibco and grown in Smooth Muscle Growth Medium-2 (SMGM-2, Lonza) at 37 °C with 5% CO_2_/95% air in 75-cm^2^ flasks. After reaching confluence, cells were washed twice with phosphate buffered saline (PBS) and the culture medium was changed to DMEM, low glucose, pyruvate (Thermo Fisher scientific) supplemented with 0.2% fetal bovine serum (Gibco) for 24 h to remove the influence of growth factors in SMGM-2 on hcSMCs. Cholesterol was then delivered to the cells by changing the medium to DMEM with water-soluble cholesterol (Sigma) at a final concentration of 1.7 μg/ml (37.5 μg/ml; cholesterol:methyl-β-cyclodextrin complexes [Chol:MβCD]). The cells were harvested at 24 h, 48 h, and 72 h after and just prior to (0 h) cholesterol loading. All experiments used hcSMCs passaged less than five times. Three biological replicates were prepared for each condition.

### 2.2 Oil red O staining and immunocytochemistry

Cells were fixed in Propylene Glycol and stained with Oil red O (Abcam, ab150678) according to the manufacturer's instructions. For immunostaining, hcSMCs were fixed in 4% paraformaldehyde in PBS and incubated with primary antibodies against human CD68 (Abcam ab955; dilution, 1:100) and α-Smooth Muscle actin (Abcam ab32575; dilution, 1:500) at 4 °C overnight. After washing in PBS, cells were incubated for 1 h at room temperature with Anti-Rabbit IgG H&L (Alexa Fluor® 555) (Abcam ab150086) and Anti-Mouse IgG H&L (Alexa Fluor® 488) (Abcam ab150117) followed by incubation with Slowfade Diamond Antifade Mountant with DAPI (Thermo Fisher Scientific S36964).

### 2.3 RNA and DNA extraction and quality assessment

Cultured hcSMCs were washed once with PBS and lysed directly with the lysis buffer in the flasks, and total RNA and DNA were extracted using RNA/DNA/Protein Purification Plus Kit (Norgen #47700). Isolated RNA was treated with DNase I (Promega) and purified with RNeasy Mini Kit (QIAGEN). The RNA samples were quantified and assessed for their integrity using the Agilent Bioanalyzer 2100 with RNA 6000 Nano kit (Agilent Technologies). The RIN (RNA integrity number) values of the RNA samples used for cDNA preparation were ≥8.0. The DNA samples was quantified using Qubit® 2.0 Fluorometer (Life technologies).

### 2.4 Quantitative reverse transcription-PCR

Total RNA (500 ng) was reverse-transcribed using SuperScript III First-strand SuperMix (Thermo Fisher Scientific) with (dT)_20_ primer. Quantitative real-time PCR were performed with TaKaRa SYBR ® Premix Ex Taq II (Tli RNaseH Plus) using StepOne Plus Instrument (Thermo Fisher Scientific). The amounts of each sample and endogenous control were determined by the relative standard curve method. The PCR protocol involved initial denaturation at 98 °C for 2 min followed by 45 thermal cycles composed of 98 °C for 10 s, 55 °C for 30 s, and 72 °C for 30 s. Primer sequences were listed in Table S1.

### 2.5 RNA-seq

We prepared RNA-seq libraries with NEBNext Ultra™ Directional RNA Library Prep Kit for Illumina (NEB, E7420) and NEBNext rRNA Depletion Kit (E6310). Library enrichment was performed by 15 cycles of PCR with NEBNext Multiplex Oligos for Illumina (NEB #E7335). Quantification of library was performed using Agilent 2100 Bioanalyzer and Kapa Library Quantification Kit (Kapa Biosystems). Libraries were sequenced with the Illumina HiSeq2000 (2 × 100 nt).

Paired-end reads were mapped to the GRCh38 reference genome using hisat2 [17]. Mapped reads were assigned to all exons using featureCounts [18]. We used the edgeR [19] Bioconductor R package to identify differentially expressed genes (DEGs) at a 1% false discovery rate (FDR) (P_adj_ ≤ 0.01) using the Benjamini–Hochberg procedure to adjust P values and with the log2 fold change (log2FC) larger than 1 (upregulated) or smaller than −1 (downregulated). Genes with <10 reads were discarded. GO enrichment analysis was performed using topGO R packages [20]. For conventional expression pattern-based clustering, DEGs were clustered into eight groups by k-means with Pearson correlation distance [21]. We determined the cluster number based on silhouette plots and scores as well as inspection of plots with K ranging from 5 to 10 so that characteristic clusters were most evidently highlighted. Motif analysis was performed using HOMER [22].

### 2.6 Methylome sequencing

We performed whole-genome bisulfite sequencing (WGBS) and Tet-assisted bisulfite sequencing (TAB-seq) with post-bisulfite adaptor tagging (PBAT) [23,24]. For the latter, we used 5hmC TAB-Seq Kit (WiseGene) to prepare DNA to be subjected to PBAT.

### 2.7 Data availability

Sequencing data were deposited in Gene Expression Omnibus (GEO) under accession number GSE163247.

## 3 Results and discussion

### 3.1 Cholesterol loading to hcSMCs

**Fig. 1:**
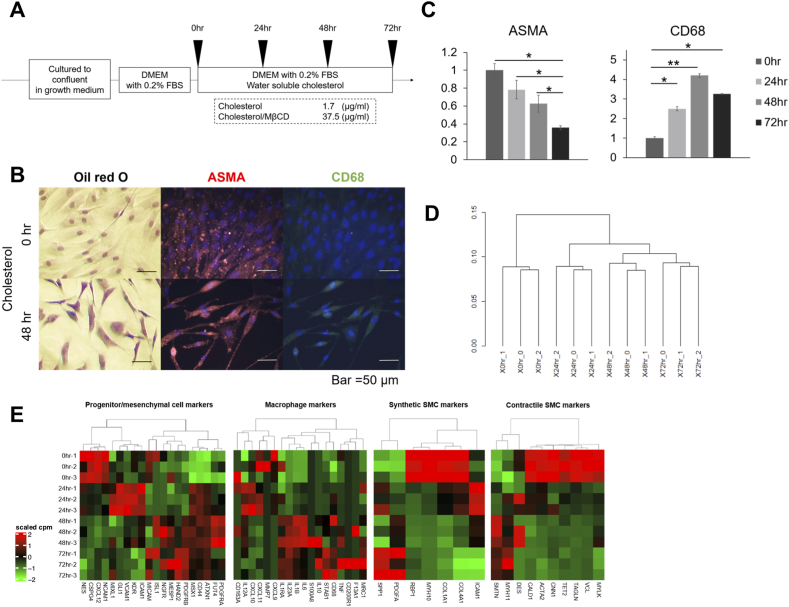
RNA-seq of cholesterol-loaded hcSMCs. (A) Schematic of cholesterol loading experiments. (B) Oil red O staining and immunostaining for ASMA and CD68 of cholesterol-loaded hcSMCs. (C) Quantitative real-time RT-PCR for ASMA and CD68 (*<0.05. **<0.01). (D) Hierarchical clustering of triplicated RNA-seq data on cholesterol-loaded hcSMCs. (E) Heatmap plots of representative gene changes in SMCs. (For interpretation of the references to color in this figure legend, the reader is referred to the Web version of this article.)

Chol:MβCD enables rapid and direct delivery of cholesterol to the plasma membrane, but excessive cholesterol in the membrane leads to cytotoxicity and cell death, as previously reported for mouse aortic SMCs [13]. Therefore, we first optimized the conditions for cholesterol loading to hcSMCs to eliminate its adverse effects, while recognizing that we had to take them into account. We consequently decided to analyze the cells at four time points (0 h, 24 h, 48 h, and 72 h) after loading of 37.5 μg/ml of Chol:MβCD (Fig. 1A and B, Supplemental Fig. 1). We used Oil red O staining to detect cholesterol accumulation in hcSMCs, which was not apparent in the first 24 h but turned out to be obvious after 48 h (Fig. 1B). At 48 h of cholesterol treatment, hcSMCs lost their original fusiform shapes and declined in their cell density. At the same time, significant decrease and increase were observed for staining of ASMA and CD68, respectively (Fig. 1B and C).

### 3.2 RNA-seq of cholesterol-loaded hcSMCs

To examine global gene expression changes during phenotype switching of hsSMCs, we performed RNA-seq at the four time points in three biological replicates. Hierarchical clustering of these 12 RNA-seq datasets confirmed that three biological replicates at each time point formed a monophyletic cluster (Fig. 1D). Confirming the reproducibility of transcriptome data, we intended to validate the biological relevance of our experimental system. For this purpose, we defined four characteristic representative gene modules based on literature review, namely contractile SMC [[25], [26], [27]], synthetic SMC [22], mesenchymal/progenitor cell [25,[28], [29], [30], [31]], and macrophage [32], and then examined their expression patterns (Fig. 1E). As expected, contractile SMC genes were rapidly down-regulated upon cholesterol-loading. Most SMC synthetic genes were also suppressed except *PDGFA* and *SPP1*. Interestingly, the mesenchymal/progenitor cell markers showed a unique mosaic pattern including transient and persistent elevation. Macrophage markers such as CD68 and interleukins were largely induced, whereas most chemokines were repressed. These results were consistent with previous findings, thus validating our experimental system.

### 3.3 Identification of pan-differentially expressed genes (pan-DEGs)

**Fig. 2:**
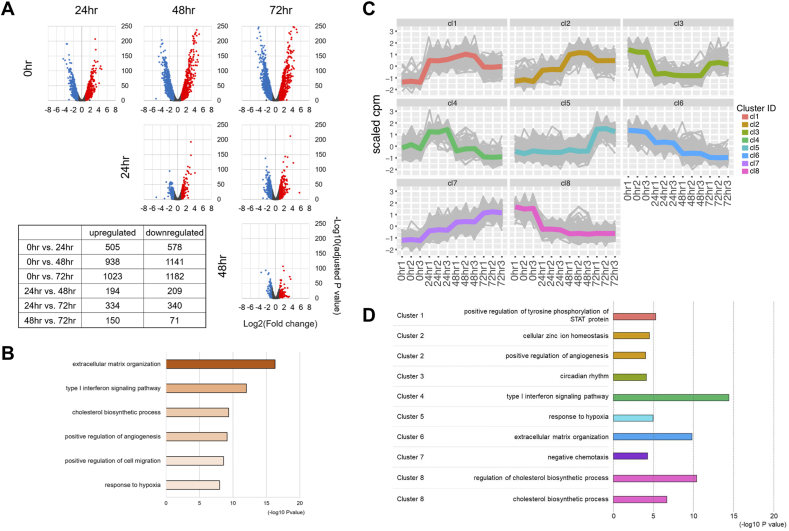
Identification and GO enrichment analysis of pan-DEGs. (A) Volcano plots for DEGs identified in all possible six combinations. Numbers of upregulated and downregulated genes are summarized in the table. (B) GO enrichment analysis of pan-DEGs. Top six GO terms are indicated with their minus log_10_(P-values). (C) Conventional k-means clustering of pan-DEGs. (D) GO enrichment analysis of clusters. Most significantly enriched GO terms were indicated for the eight clusters identified in (C). GO terms identified in (B) are also indicated.

We next intended to identify DEGs. Note that we performed pairwise comparison not only between the consecutive time points but also between all the other possible combinations. This was because comparison between every possible pairwise combination should ensure exhaustive identification of DEGs. Volcano plots and the number of upregulated and downregulated genes were shown for all of the six possible combinations between the four time points (Fig. 2A). We then removed the overlaps among the six DEG sets to define a non-redundant set composed of 2978 genes in total (Table S3). Hereinafter we call them pan-DEGs.

Among the pan-DEGs, 505 and 194 were upregulated between 0 h and 24 h and between 24 h and 48 h, respectively (Fig. 2A). Although the sum of these two DEG sets could be 699 at its maximum, it fell far short of 938 upregulated DEGs identified between 0 h and 48 h (Fig. 2A). This was because expression changes of a substantial number of genes were significant between 0 h and 48 h but insignificant between 0 h and 24 h and between 24 h and 48 h. Such gradually induced genes would have been missed in the comparison between consecutive time points, underscoring the importance of exhaustive comparison to define pan-DEGs.

We used the pan-DEGs in GO enrichment analysis to maximize biological implications deduced from the transcriptome changes. Enriched GO terms included “extracellular matrix organization”, “type I interferon signaling pathway”, “cholesterol biosynthetic process”, “positive regulation of angiogenesis”, “positive regulation of cell migration”, and “response to hypoxia” (Fig. 2B, Table S4).

Next, we applied the conventional k-means clustering to the pan-DEGs and grouped them into eight clusters (Fig. 2C). Genes in each cluster were then subjected to GO enrichment analysis (Fig. 2D). Among the six GO terms enriched in pan-DEGs (Fig. 2B), we readily identified “extracellular matrix organization”, “type I interferon signaling pathway”, “cholesterol biosynthetic process”, “positive regulation of angiogenesis”, and “response to hypoxia” as GO terms enriched with high significance in clusters 6, 4, 8, 2, and 5, respectively (Fig. 2D, Table S5). In contrast, the other GO term “positive regulation of cell migration” was not enriched in any of the eight clusters (Table S5). This was because 126 pan-DEGs associated with this GO term were scattered throughout the eight clusters (13, 25, 4, 5, 7, 13, 18, and 41 genes in clusters 1 to 8, respectively) and because each scattered subset is diluted by many other genes in each cluster, failing to demonstrate significant enrichment of “positive regulation of cell migration” (Table S5). Note that some subsets of these genes enriched other GO terms. For instance, the 126 pan-DEGs included 17 genes assigned to the GO term “semaphorin-plexin signaling pathway”. Of these 17 genes, seven and other seven were included in clusters 2 and 8, respectively, thus displaying distinct expression patterns. Consequently, this GO term was enriched in these two clusters (cluster 2, p = 8.4E-05; cluster 8, p = 8.5E-04, Table S5) albeit less prominently than it was in pan-DEGs (p = 9.7E-09, Table S4).

These examples illustrated potential pitfalls in interpreting time-course RNA-seq data with a conventional expression pattern-based clustering scheme. First, if genes of many distinct functional categories exhibit a similar expression pattern to form a single cluster, then each functional category is diluted in the cluster and tends to be overlooked in the enrichment analysis. However, GO terms enriched in pan-DEGs would attract attention even if its enrichment in a cluster is not so prominent. For instance, the GO terms “response to hypoxia” and “negative chemotaxis” were enriched in clusters 5 and 7, respectively, but the enrichment in these clusters was less prominent compared to the enrichment in pan-DEGs (Tables S4 and S5). Second, since even genes sharing the same GO term may exhibit distinct expression dynamics, biological interpretation should be refined by integrating the expression patterns (see below).

### 3.4 GO-centric clustering of pan-DEGs

**Fig. 3:**
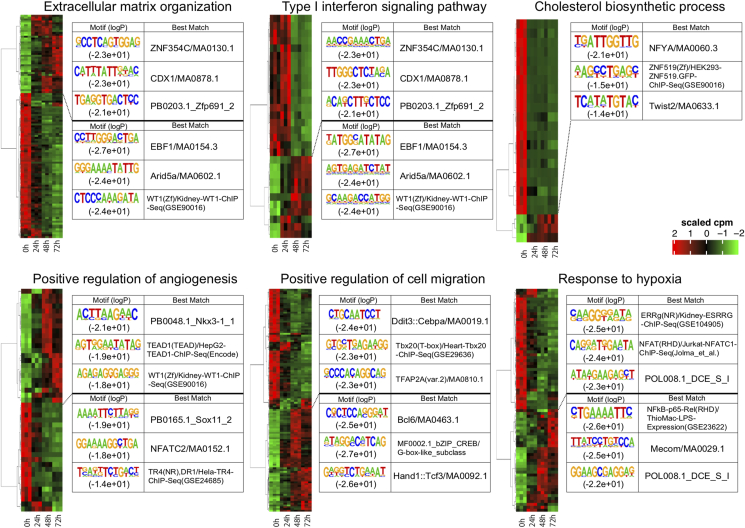
GO-centric clustering of pan-DEGs. DEGs associated with each of the six GO terms in Fig. 2B are subjected to hierarchical clustering. TFBS motifs enriched for the top two subclusters are also indicated. Note that DEGs associated with the same GO term showed distinct patterns in expression and motif enrichment.

The results described above prompted us to take an alternative approach for biological interpretation of DEGs. In this approach, we first applied GO enrichment analysis to pan-DEGs (Fig. 2B) and then subjected DEGs associated with each GO term to hierarchical clustering. We termed this approach as “GO-centric” clustering. Heat maps of GO-centric clustering clearly indicated that DEGs associated to each enriched GO term did not share a similar expression pattern but rather exhibited two or more distinct patterns to form subclusters (Fig. 3). GO-centric clustering would thus prevent its users from overlooking multifaceted responses of genes sharing the same GO term.

To address the mechanisms underlying distinct responses among DEGs sharing the same GO, we examined the enrichment of transcription factor-binding site (TFBS) motifs in their promoter regions. As expected, different TFBS motifs were enriched between the two top subclusters, often representing upregulated and downregulated DEGs (Fig. 3). For instance, promoters of the upregulated subcluster of DEGs associated with “extracellular matrix organization” significantly enriched a TFBS motif for CDX1, which regulates epicardial epithelial-to-mesenchymal transition and the migration and differentiation of epicardium-derived progenitors into vascular smooth muscle cells [34] (Fig. 3). In contrast, promoters of the downregulated subcluster enriched a TFBS motif for Arid5a (AT-rich interactive domain-containing protein 5a) known to stabilize a variety of inflammatory mRNA transcripts such as Il-6 and Stat3 and contribute to the inflammatory response [35] (Fig. 3). These findings would provide potentially interesting clues to mechanistically dissect reorganization of extracellular matrix associated with cholesterol-induced transdifferentiation of hcSMCs.

Taken together, GO-centric clustering based on GO enrichment analysis of pan-DEGs would provide us with unique opportunities to interrogate the details and potential mechanisms of differential gene expression. It would thus complement the conventional expression pattern-based clustering to provide alternative points of view in interpretating complex transcriptome data, thereby enabling deeper interpretation of time-course transcriptome data.

### 3.5 Methylation and hydroxymethylation patterns among pan-DEGs

**Fig. 4:**
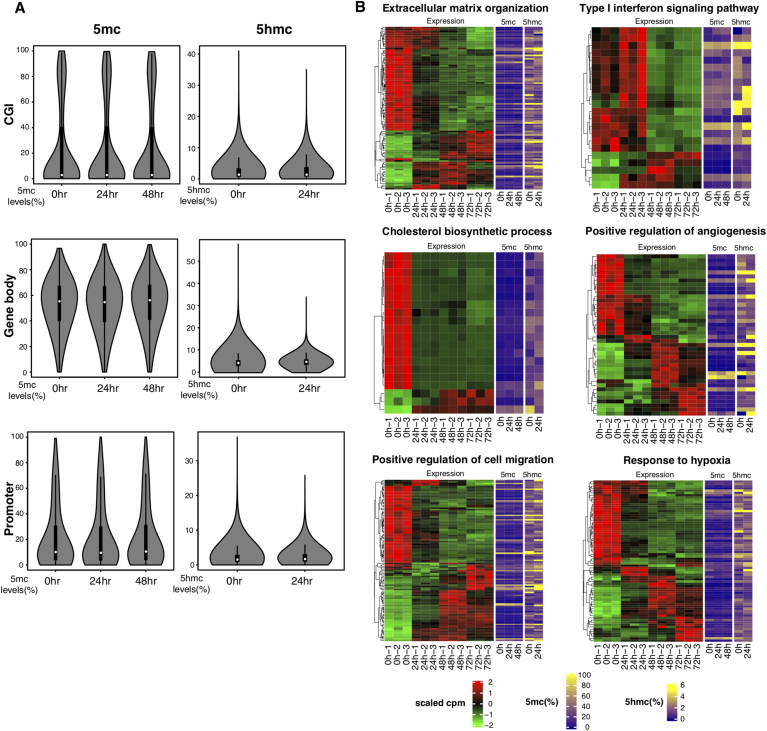
(Hydroxy)methylome alterations in cholesterol-loaded hcSMCs. (A) Violin plots. Left panels, methylation (5 mC) levels of CGI, gene body, and promoter at 0 h, 24 h, and 48 h. Right panels, hydrocymethylation (5hmC) levels of CGI, gene body, and promoter at 0 h and 24 h. (B) Promoter methylation (5 mC) and hydroxymethylation (5hmC) levels (yellow-violet color scale) and expression levels (green-red color scale) of DEGs associated with the six GO terms identified in Fig. 2B. (For interpretation of the references to color in this figure legend, the reader is referred to the Web version of this article.)

As a potential mechanism regulating differential gene expression, we examined (hydroxy)methylomes because SMC plasticity was reported to be regulated by TET2 involved in DNA demethylation via hydroxymethylation [26,36]. We performed WGBS on hcSMCs at 0 h, 24 h, and 48 h. However, WGBS did not reveal any obvious changes in methylation levels of CpG island, gene body, and promoter (Fig. 4A). We also performed TAB-seq on hcSMCs at 0 h and 24 h. Levels of methylation and hydroxymethylation deduced from WGBS and TAB-seq data did not show significant changes in CpG island between 0 h and 24 h. Although gene body and promoter showed slightly lower hydroxymethylation levels at 24 h than 0 h, the differences were not statistically significant. These results indicated that no global (hydorxy)methylomic change occurred during cholesterol loading.

We next compared the promoter (hydroxy)methylation levels of DEGs classified by GO-centric clustering (Fig. 4B). Downregulated DEGs involved in “type I interferon signaling pathway,” “positive regulation of angiogenesis,” and “response to hypoxia” had higher overall hydroxymethylation levels than the other DEG groups. However, (hydroxy)methylation changstes during transdifferentiation were limited, and the genomic regions with differential (hydroxy)methylation largely failed to fall in the vicinity of DEGs. We thus assumed that (hydroxy)methylation changes have only a marginal, if any, effect on the observed transcriptome alterations. These results do not contradict with the previous findings that the correlation between DNA methylation and gene expression can be both positive and negative [37] and both hyper- and hypomethylation can be associated with cardiovascular diseases [38]. Further studies are required to unveil the roles of DNA hyper- and hypomethylation in pathology of atherosclerosis.

### 3.6 Conclusions

We performed a time-course RNA-seq analysis on cholesterol-loaded hcSMCs and exhaustively identified DEGs through all possible pairwise comparison between the time points to define pan-DEGs. GO-centric clustering of pan-DEGs enabled us to pay attention to such alterations that would otherwise escape our attention, suggesting its general utility in interpretating time-course transcriptome data. Transient induction of type I interferon response may be of particular interest in the light of recent attentions to its roles in atherosclerosis [39], especially, in terms of its coupling with cholesterol metabolism [33]. We expect the datasets would provide an invaluable resource for studies on atherogenesis.

## Declaration of competing interest

The authors declare that they have no known competing financial interests or personal relationships that could have appeared to influence the work reported in this paper.
