# Supplementary material for: Transcriptome dynamics during cholesterol-induced transdifferentiation of human coronary artery smooth muscle cells: A Gene Ontology-centric clustering approach
Source: Biochem Biophys Rep. 2021 Jun 27;27:101061. doi: 10.1016/j.bbrep.2021.101061 (PMC8254084; doi:10.1016/j.bbrep.2021.101061)
Supplement: Multimedia component 2 [file mmc2.pdf]

Supplemental figure

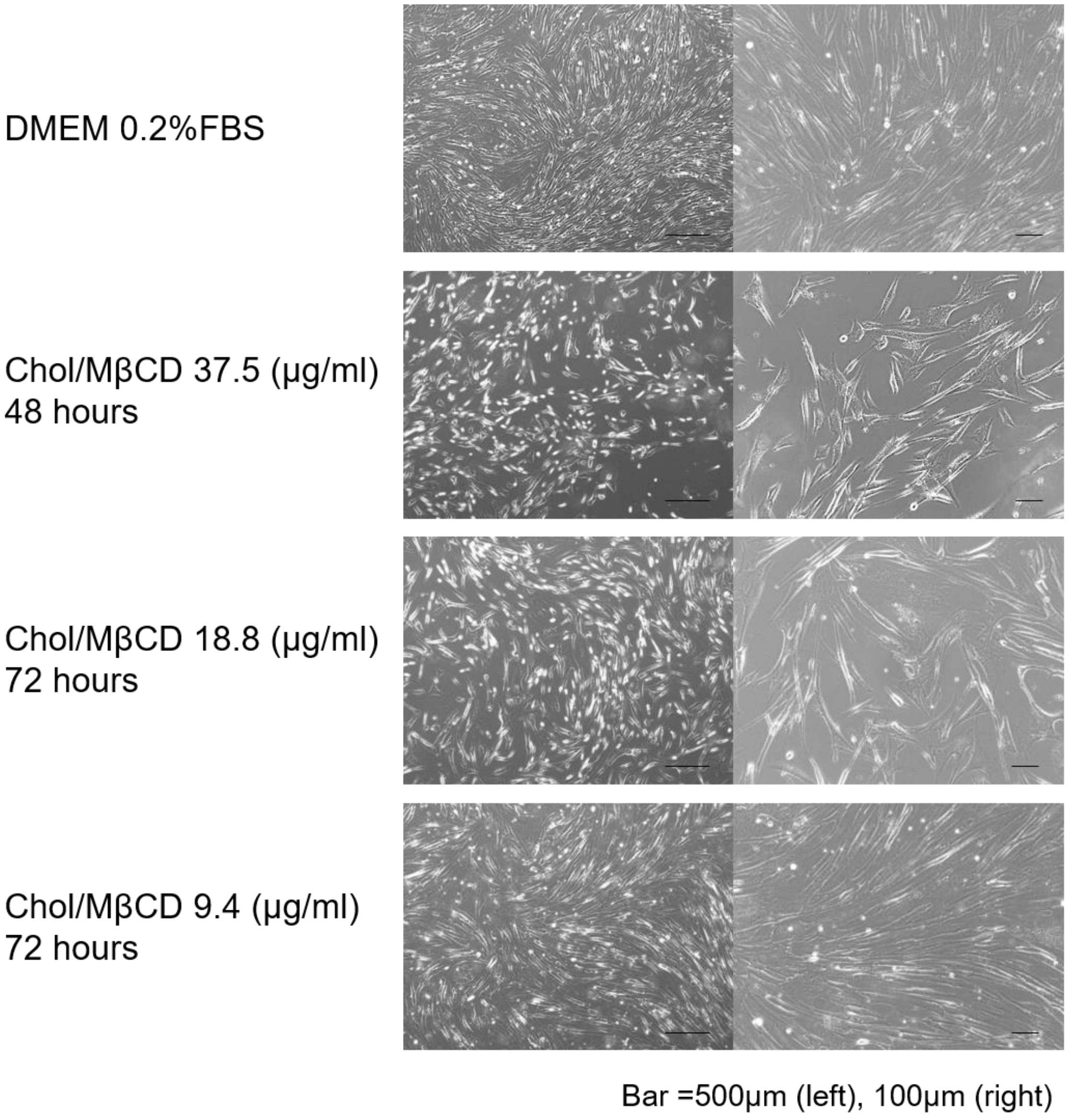

Supplemental Figure 1: Establishment of a robust cholesterol loading protocol to explore the phenotype switching process of hcSMCs.

Cholesterol/MβCD less than 37.5 μg/ml failed to evoke noticeable morphological changes in hcSMCs even with 72 hour-incubation. On the other hand, most hcSMCs could not tolerate with >72 hour-incubation with cholesterol/MβCD more than 37.5 μg/ml (data not shown). We thus decided to evaluate the cells at four time points or every 24 hours (i.e., 0, 24, 48, and 72 hours) after loading of 37.5 μg/ml of cholesterol (Fig. 1A and B).
